# Adherence to Cancer Prevention Lifestyle Recommendations Before, During, and 2 Years After Treatment for High-risk Breast Cancer

**DOI:** 10.1001/jamanetworkopen.2023.11673

**Published:** 2023-05-04

**Authors:** Rikki A. Cannioto, Kristopher M. Attwood, Evan W. Davis, Lucas A. Mendicino, Alan Hutson, Gary R. Zirpoli, Li Tang, Nisha M. Nair, William Barlow, Dawn L. Hershman, Joseph M. Unger, Halle C. F. Moore, Claudine Isaacs, Timothy J. Hobday, Gabriel N. Hortobagyi, Julie R. Gralow, Kathy S. Albain, G. Thomas Budd, Christine B. Ambrosone

**Affiliations:** 1 Department of Cancer Prevention & Control, Roswell Park Comprehensive Cancer Center, Buffalo, New York; 2 Department of Biostatistics and Bioinformatics, Roswell Park Comprehensive Cancer Center, Buffalo, New York; 3 Slone Epidemiology Center, Boston University, Boston, Massachusetts; 4 Southwest Oncology Group Statistics and Data Management Center, Fred Hutchinson Cancer Center, University of Washington, Seattle; 5 Herbert Irving Comprehensive Cancer Center at Columbia University, New York, New York; 6 Department of Hematology and Medical Oncology, Cleveland Clinic, Cleveland, Ohio; 7 Lombardi Comprehensive Cancer Center, Georgetown University, Washington, DC; 8 Department of Oncology, Mayo Clinic College of Medicine, Rochester, Minnesota; 9 Department of Breast Medical Oncology, Division of Cancer Medicine, The University of Texas MD Anderson Cancer Center, Houston; 10 Fred Hutchinson Cancer Center and the Seattle Cancer Care Alliance, University of Washington, Seattle-; 11 Division of Hematology/Oncology, Cardinal Bernardin Cancer Center, Loyola University Chicago Stritch School of Medicine, Chicago, Illinois

## Abstract

**Importance:**

The American Institute for Cancer Research and American Cancer Society regularly publish modifiable lifestyle recommendations for cancer prevention. Whether these recommendations have an impact on high-risk breast cancer survival remains unknown.

**Objective:**

To investigate whether adherence to cancer prevention recommendations before, during, and 1 and 2 years after breast cancer treatment was associated with disease recurrence or mortality.

**Design, Setting, and Participants:**

The Diet, Exercise, Lifestyles, and Cancer Prognosis Study (DELCaP) was a prospective, observational cohort study designed to assess lifestyles before diagnosis, during treatment, and at 1 and 2 years after treatment completion, implemented ancillary to the Southwest Oncology Group (SWOG) S0221 trial, a multicenter trial that compared chemotherapy regimens in breast cancer. Participants were chemotherapy-naive patients with pathologic stage I to III high-risk breast cancer, defined as node-positive disease with hormone receptor–negative tumors larger than 1 cm or any tumor larger than 2 cm. Patients with poor performance status and comorbidities were excluded from S0221. The study was conducted from January 1, 2005, to December 31, 2010; mean (SD) follow-up time for those not experiencing an event was 7.7 (2.1) years through December 31, 2018. The analyses reported herein were performed from March 2022 to January 2023.

**Exposure:**

An aggregated lifestyle index score comprising data from 4 time points and 7 lifestyles, including (1) physical activity, (2) body mass index, (3) fruit and vegetable consumption, (4) red and processed meat intake, (5) sugar-sweetened beverage consumption, (6) alcohol consumption, and (7) smoking. Higher scores indicated healthier lifestyle.

**Main Outcomes and Measures:**

Disease recurrence and all-cause mortality.

**Results:**

A total of 1340 women (mean [SD] age, 51.3 [9.9] years) completed the baseline questionnaire. Most patients were diagnosed with hormone-receptor positive breast cancer (873 [65.3%]) and completed some education beyond high school (954 [71.2%]). In time-dependent multivariable analyses, patients with highest vs lowest lifestyle index scores experienced a 37.0% reduction in disease recurrence (hazard ratio, 0.63; 95% CI, 0.48-0.82) and a 58.0% reduction in mortality (hazard ratio, 0.42; 95% CI, 0.30-0.59).

**Conclusions and Relevance:**

In this observational study of patients with high-risk breast cancer, strongest collective adherence to cancer prevention lifestyle recommendations was associated with significant reductions in disease recurrence and mortality. Education and implementation strategies to help patients adhere to cancer prevention recommendations throughout the cancer care continuum may be warranted in breast cancer.

## Key Points

**Question:**

Is adherence to cancer prevention lifestyle recommendations before, during, and after chemotherapy associated with disease recurrence and mortality in patients with high-risk breast cancer?

**Findings:**

In this prospective cohort study of 1340 patients with high-risk breast cancer, strongest adherence to the American Cancer Society and American Institute of Cancer Research prevention recommendations was associated with a 37% reduced hazard of breast cancer recurrence and a 58% reduced hazard of mortality.

**Meaning:**

These findings suggest that education and implementation strategies to help patients adhere to cancer prevention recommendations throughout the cancer care continuum may be warranted in breast cancer.

## Teaser

This cohort study investigates whether adherence to overlapping cancer prevention lifestyle recommendations before, during, and after treatment was associated with recurrence or mortality among patients with high-risk breast cancer.

## Introduction

The American Institute for Cancer Research (AICR) and the American Cancer Society (ACS) regularly publish cancer prevention recommendations for decreasing the risk of developing cancer.^1,2^ The most recent recommendations include (1) maintaining a healthy body weight; (2) meeting the physical activity (PA) guidelines; (3) eating a colorful variety of vegetables, fruits, and plenty of whole grains; (4) limiting red and processed meats, fast food, and other highly processed food; (5) avoiding or limiting sugar-sweetened beverages; (6) avoiding or limiting alcohol consumption to 1 drink or fewer per day; and (8) avoiding cigarette smoking.^1,2,3,4^ Despite recommendations to adhere to prevention guidelines after a cancer diagnosis, which lifestyle factors have an impact on cancer outcomes, and whether those factors work together, remains unknown.

Recently, a National Cancer Institute collaborative group published guidance for developing analytic approaches to address this gap in knowledge.^3,4^ The resultant work encourages researchers to implement a standardized lifestyle score to investigate how adherence to prevention recommendations may impact outcomes, including cancer mortality.^3,4^

To date, epidemiologic evidence supports an association for some, but not all, individual lifestyle recommendations with breast cancer (BC) survival.^5,6,7,8,9,10,11,12,13,14,15,16,17^ However, because many lifestyle behaviors co-occur, investigations of independent behaviors may ignore cumulative effects that could impact recurrence or mortality.^18^ Thus, an aggregate lifestyle score may better reflect whether adhering to cancer prevention recommendations is also associated with BC outcomes.^19^ In accordance with National Cancer Institute guidance,^3,4^ we created a lifestyle index score (LIS) to investigate whether adherence to overlapping AICR and ACS recommendations before, during, and after treatment was associated with recurrence or mortality among patients with high-risk BC enrolled in the Diet, Exercise, Lifestyles, and Cancer Prognosis (DELCaP) Study.

## Methods

### Study Population and Data Collection

**Figure 1.**
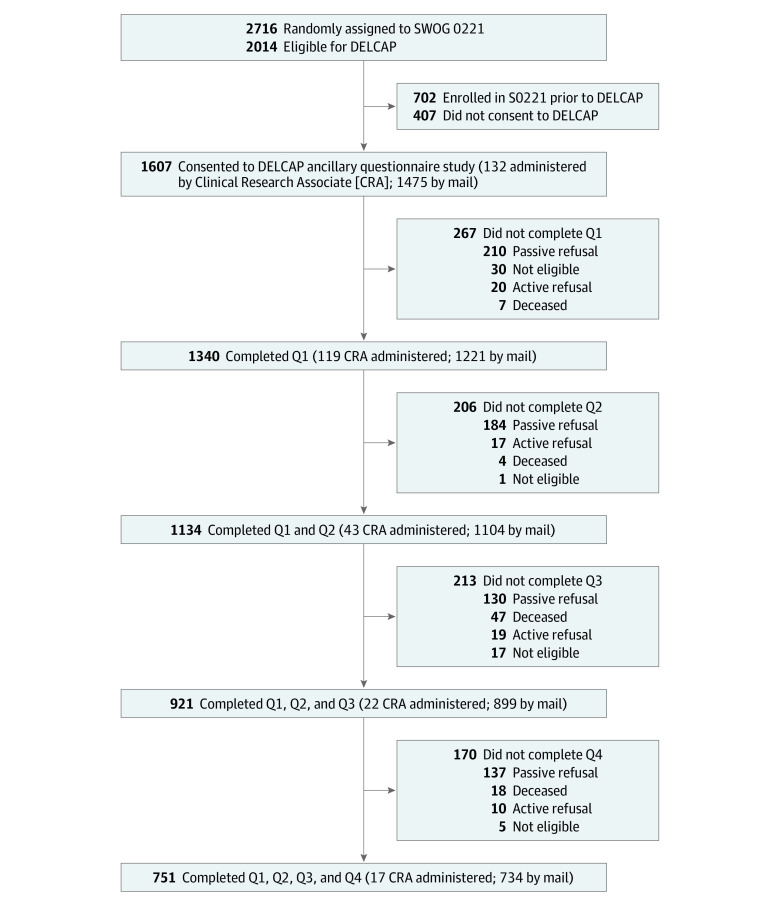
The Diet, Exercise, Lifestyles, and Cancer Prognosis (DELCaP) Study Schema Participants included in the DELCaP Study, a prospective, observational questionnaire study ancillary to the Southwest Oncology Group (SWOG) S0221 trial, a randomized treatment trial for high-risk breast cancer. Questionnaire 1 (Q1) was completed at the time of S0221 registration, before the initiation of chemotherapy, and represents lifestyles in the 4 weeks before diagnosis. Questionnaire 2 (Q2) was completed at the completion of active treatment (approximately 6 months after trial registration) and represents lifestyles during treatment. Questionnaire 3 (Q3) was completed 1 year after trial registration and represents lifestyles during the previous year. Questionnaire 4 (Q4) was completed 2 years after trial registration and represents lifestyles in the previous year. Patients who did not return subsequent questionnaires but did not formally withdraw from the study were identified as passive refusals; patients withdrawing from study were identified as active refusals.

The DELCaP Study was a prospective, observational cohort study ancillary to a multicenter phase 3 clinical trial led by the Southwest Oncology Group (SWOG) (S0221; NCT00070564)^20^ that randomly assigned patients with high-risk stage I to III BC to different treatment schedules with doxorubicin, cyclophosphamide, and paclitaxel. DELCaP was initiated to assess the role of lifestyle factors before, during, and after treatment in relation to BC outcomes.^21,22,23^ Details regarding enrollment, inclusion, and exclusion criteria for S0221 have been previously described.^21^ Briefly, patients were excluded if they received prior systemic therapy or had comorbidities, abnormal organ function, or poor performance status. Patients experiencing toxicities or treatment delays greater than 3 weeks were removed from the trial. Approval to initiate DELCaP was obtained from the institutional review boards at Roswell Park and at all participating institutions that enrolled patients in S0221. The study was conducted from January 1, 2005, to December 31, 2010; mean (SD) follow-up time among patients not experiencing an event was 7.7 (2.1) years through December 31, 2018. A total of 2014 patients from S0221 were eligible to participate in DELCaP; 1607 (70.8%) provided written informed consent, and 1340 (83.4%) completed the baseline questionnaire. Response rates for each subsequent questionnaire and reasons for loss to follow-up are shown in Figure 1. This report follows the Strengthening the Reporting of Observational Studies in Epidemiology (STROBE) reporting guideline for cohort studies.^24^

### The DELCaP Questionnaire

A detailed description of the DELCaP questionnaire has been previously published.^21^ The questionnaire was adapted from the VITAL Study, which was extensively validated.^23^ The baseline questionnaire (Q1) was administered at enrollment and queried lifestyles 1 month before diagnosis. Questionnaire 2 (Q2) was administered at the time of treatment completion to patients completing Q1 and assessed lifesyles during treatment. Questionnaire 3 (Q3) was administered to patients completing Q1 and Q2 approximately 1 year after study enrollment and assessed lifestyles in the preceding year. Questionnaire 4 (Q4) was administered 2 years after study enrollment to patients completing Q1 to Q3 and queried lifestyles in the preceding year. Self-identified race and ethnicity were queried using the DELCaP questionnaire. For both questionnaire items assessing race and ethnicity, respondents were instructed to mark all that apply, which included options for “other” and “don’t know.” Throughout the study, participants submitting surveys with missing responses were contacted by Clinical Research Associates to maximize completeness.

### Lifestyle Assessment and Scoring

We created a LIS^3,4^ that reflected lifestyle adherence at 4 time points to the following 7 cancer prevention recommendations: (1) aim to meet the PA guidelines, (2) maintain a normal body mass index (BMI; calculated as weight in kilograms divided by height in meters squared), (3) increase consumption of a colorful variety of fruit and vegetables, (4) limit consumption of red and processed meat, (5) limit sugar-sweetened beverage consumption, (6) avoid alcohol, and (7) avoid smoking. For each lifestyle, a score of 1 point represented strongest adherence, a half point represented partial adherence, and a zero represented nonadherence.^3,4^ To create the LIS at each time point, adherence scores for each lifestyle were summed, with total scores ranging from 0 (nonadherence) to 7 (strongest adherence).^3,4^ To account for lifestyle changes over time, an aggregated time-varying LIS was calculated, comprising data from Q1 through Q4, and served as the primary exposure of interest. For all multivariable analyses, lifestyle adherence scores were categorized into tertiles, with the highest tertile reflecting strongest adherence. Detailed methods for assessing individual lifestyles and calculating and parameterizing adherence scores are provided in the eMethods in Supplement 1.

### Clinical Outcome Ascertainment

The primary analytic outcomes of interest were hazards of disease recurrence and all-cause mortality. For recurrence, survival time included time from randomization to first instance of disease recurrence, new breast primary tumor, or death from any cause, whichever came first. Recurrence was assessed via physical examination every 6 months for 5 years and annually for up to 15 years or until death. For mortality, survival time included time from randomization to death from any cause. Vital status was ascertained from medical records, patient and family contact, obituaries, and national indexes. Patients without disease recurrence and those who were still alive at the time of analysis were censored on the date of their last clinical contact.

### Statistical Analysis

The analyses reported herein were performed from January 2022 to March 2023. First, we examined univariable associations of patient characteristics with BC outcomes and the aggregate LIS. Second, Kaplan-Meier curves were generated to characterize the disease-free and overall survival experience according to the baseline LIS. Third, in primary multivariable analyses, time-dependent Cox proportional hazards regression models were used to assess associations of the aggregated LIS with BC outcomes. Considering the LIS as a time-varying exposure appropriately accounted for changes in lifestyles throughout the study while also accounting for the possibility of immortal time bias.^25,26^ Fourth, in secondary multivariable analyses, we used standard Cox proportional hazards regression models to assess associations of the LIS with BC outcomes at each time point. For Q2 to Q4, we conducted landmark analyses to further account for immortal time bias. However, because the landmark time became shorter with each successive questionnaire and data points were lost, these analyses provide an incomplete representation of the exposure-outcome association.^26^ Fifth, to assess the contribution of each individual lifestyle in the aggregated LIS-outcome association at each time point, we conducted time-dependent leave-out analyses. To accomplish this, we excluded each lifestyle factor from the aggregated LIS at each time point and quantified the magnitude and direction of change in the observed association with BC outcomes using the remaining 6 factors.^18^

#### Assessment of Confounding

For all multivariable analyses, we a priori defined age at baseline and a stratification factor corresponding to treatment assignment from S0221 as important covariates. Next, we applied well-established conceptual and empirical methods for identifying additional confounders as described in the eMethods in Supplement 1. Briefly, we used the definition of confounding,^27^ directed acyclic graphs (eFigure 1 in Supplement 1), the change-in-estimate method,^28^ and stepwise regression^29^ to identify confounders for adjustment in multivariable models.

Despite meeting conceptual criteria of confounding, adjustment for self-identified race, self-identified ethnicity, educational attainment, and menopause status did not change estimates of association, and these factors were not statistically significant factors in stepwise regression. Conversely, the number of positive nodes, *ERBB2* (previously *HER2/neu*) (OMIM 164870) status, and *ER* (OMIM 620207)*/PGR* (OMIM 607311) status were statistically significant factors. Thus, fully adjusted multivariable models included age at study enrollment, number of positive nodes, *ERBB2* and *ER/PGR* status, and a stratification factor for treatment arm.

To detect departures from model assumptions that may have influenced our estimates, we used standard diagnostic methods, including examining residuals, ad hoc time-varying covariates of a discretized time scale, and Kaplan-Meier curves. For patients with 1 or more missing surveys, data were classified as missing from nonresponse and were assumed to be missing not at random. As previously described,^21^ Taylor series variance estimation was used to account for missing data; observations with missing values were included in computing the degrees of freedom.^30^

#### Sensitivity Analyses

We conducted a series of sensitivity analyses to assess the potential role of selection bias or confounding in our estimates. First, to assess potential selection effects into DELCaP, we compared 5-year survival of all patients enrolled in S0221 with patients enrolled in DELCaP. Second, to examine the possibility that lifestyles were associated with questionnaire response rates, we examined the association of LIS with nonresponse at each time point. Third, to quantitatively assess potential bias from unmeasured confounding, we calculated the E-value.^31^ Fourth, we examined the potential role of BC subtype, menopause status, self-identified race, and educational attainment as effect modifiers of the primary exposure–outcome association.

All statistical tests were 2-sided, and *P* < .05 was considered statistically significant. All analyses were performed using SAS software, version 9.4 (SAS Institute Inc).

## Results

A total of 1340 women (mean [SD] age, 51.3 [9.9] years) enrolled in DELCaP and completed the baseline questionnaire (Table); most participants were postmenopausal (696 [52.5%]), self-identified as non-Hispanic White (1118 [83.7%]), completed at least some college education (954 [71.2%]), and were diagnosed with hormone-receptor positive BC (873 [65.3%]). During a mean (SD) follow-up time of 7.7 (2.1) years, 310 events of disease progression (23.1%) and 222 events of death (16.6%) occurred.

**Table.** Epidemiologic and Clinical Characteristics at Baseline in the Overall DELCaP Study Population and According to Disease Recurrence and Mortality^a^

Characteristic	Overall, No. (%) (N = 1340)^b^	Disease recurrence			Mortality		
		Disease free (n = 1030 [76.9%])	Recurrence (n = 310 [23.1%])	*P* value^c^	Alive (n = 118 [83.4%])	Deceased(n = 222 [16.6%])	*P* value^c^
Recurrence time, mean (SD), mo	81.1 (34.1)	92.5 (26.1)	43.3 (30.2)	<.001	NA	NA	NA
Survival time, mean (SD), mo	86.1 (30.9)	NA	NA	NA	92.9 (26.0)	51.8 (31.0)	<.001
Age at enrollment, mean (SD), y	51.3 (9.9)	50.9 (9.6)	52.8 (10.6)	.005	50.9 (9.7)	53.4 (10.5)	.001
Educational attainment							
No high school diploma	93 (7.0)	61 (6.0)	32 (10.3)	.06	69 (6.2)	24 (10.8)	.03
High school graduate or GED	287 (21.5)	219 (21.4)	68 (21.9)		234 (21.0)	53 (23.9)	
Some college	484 (36.3)	380 (37.1)	104 (33.5)		412 (37.1)	72 (32.4)	
College graduate	286 (21.4)	227 (22.2)	59 (19.0)		248 (22.3)	38 (17.1)	
Advanced degree	184 (13.8)	137 (13.4)	47 (15.2)		149 (13.4)	35 (15.8)	
Race							
African American/Black	94 (7.0)	64 (6.2)	30 (9.7)	.496	69 (6.2)	25 (11.3)	.13
American Indian	13 (1.0)	10 (1.0)	3 (1.0)		11 (1.0)	2 (0.9)	
Asian	43 (3.2)	36 (3.5)	7 (2.3)		40 (3.6)	3 (1.4)	
Multiracial	45 (3.4)	35 (3.4)	10 (3.2)		38 (3.4)	7 (3.2)	
Pacific Islander	5 (0.4)	4 (0.4)	1 (0.3)		4 (0.4)	1 (0.5)	
Other^d^	18 (1.4)	14 (1.4)	4 (1.3)		15 (1.3)	3 (1.4)	
White	1118 (83.7)	864 (84.1)	254 (82.2)		937 (84.1)	181 (81.5)	
Ethnicity							
Hispanic	65 (4.9)	46 (4.5)	19 (6.1)	.23	54 (4.8)	11 (5.0)	.94
Non-Hispanic	1275 (95.2)	984 (95.5)	291 (93.9)		1064 (95.2)	211 (95.0)	
Menopause status							
Premenopausal	630 (47.5)	506 (49.6)	124 (40.7)	.006	545 (49.2)	85 (39.0)	.006
Postmenopausal	696 (52.5)	515 (50.4)	181 (59.3)		563 (50.8)	133 (61.0)	
Lymph node classification							
Node negative	349 (26.1)	290 (28.2)	59 (19.0)	<.001	309 (27.7)	40 (18.0)	<.001
1-3 Positive nodes	502 (37.5)	406 (39.5)	96 (31.0)		433 (38.8)	69 (31.1)	
≥4 Positive nodes	487 (36.4)	332 (32.3)	155 (50.0)		374 (33.5)	113 (50.9)	
*ERBB2* status							
Negative	1055 (79.1)	793 (77.4)	262 (84.5)	.007	867 (78.0)	188 (84.7)	.02
Positive	279 (20.9)	231 (22.6)	48 (15.5)		245 (22.0)	34 (15.3)	
Hormone receptor status							
Negative	464 (34.7)	338 (32.9)	126 (40.8)	.01	365 (32.7)	99 (44.8)	<.001
Positive	873 (65.3)	690 (67.1)	183 (59.2)		751 (67.3)	122 (55.2)	
Tumor subtype							
*ERBB2* positive	279 (21.0)	231 (22.6)	48 (15.5)	.003	245 (22.1)	34 (15.4)	.004
*ERBB2* negative and hormone receptor positive	703 (52.8)	542 (53.0)	161 (52.1)		592 (53.3)	111 (50.2)	
Triple negative (*ERBB2* and hormone receptor negative)	350 (26.3)	250 (24.4)	100 (32.4)		274 (24.7)	76 (34.4)	
Physical activity^e^							
0 (inactive; no MVPA)	359 (26.8)	290 (25.9)	69 (31.1)	.046	265 (25.7)	94 (30.3)	.14
0.5 (insufficient MVPA; <7.5 MET h/wk)	330 (24.6)	268 (24.0)	62 (27.9)		250 (24.3)	80 (25.8)	
1 (meeting guidelines; ≥7.5 MET h/wk)	651 (48.6)	560 (50.1)	91 (41.0)		515 (50.0)	136 (43.9)	
BMI (continuous), mean (SD)	29.0 (6.6)	28.8 (6.5)	29.7 (7.2)	.11	28.8 (6.5)	30.0 (7.2)	.054
0 (underweight [<18.5] or obesity [≥30])	497 (37.7)	408 (37.1)	89 (40.6)	.24	373 (36.8)	124 (40.4)	.36
0.5 (overweight [25.0-29.99])	435 (33.0)	359 (32.6)	76 (34.7)		333 (32.9)	102 (33.2)	
1 (normal weight [18.5-24.9])	388 (29.4)	334 (30.3)	54 (24.7)		307 (30.3)	81 (26.4)	
Fruit and vegetable intake^f^							
0 (lowest intake [<20.75 SPW])	446 (33.3)	368 (32.9)	78 (35.1)	.80	336 (32.6)	110 (35.5)	.64
0.5 (middle tertile [20.75-36.85 SPW])	446 (33.3)	373 (33.4)	73 (32.9)		346 (33.6)	100 (32.3)	
1 (highest intake [>36.85 SPW])	448 (33.4)	377 (33.7)	71 (32.0)		348 (33.8)	100 (32.3)	
Processed meat intake							
0 (highest intake [>7.75 SPW])	453 (33.8)	367 (32.8)	86 (38.7)	.16	344 (33.4)	109 (35.2)	.51
0.5 (middle tertile [4.00-7.75 SPW])	444 (33.1)	371 (33.2)	73 (32.9)		337 (32.7)	107 (34.5)	
1 (lowest intake [<4.00 SPW])	443 (33.1)	380 (34.0)	63 (28.4)		349 (33.9)	94 (30.3)	
Sugar-sweetened beverage^g^							
0 (highest intake [≥16.5 SPM])	455 (34.0)	379 (34.0)	76 (34.2)	.11	351 (34.1)	104 (33.5)	.62
0.5 (middle tertile [3.0-16.4 SPM])	450 (33.6)	364 (32.6)	86 (38.7)		339 (33.0)	111 (35.8)	
1 (lowest intake [<3.0 SPM])	433 (32.4)	373 (33.4)	60 (27.0)		338 (32.9)	95 (30.6)	
Smoking status							
0 (current smoker)	171 (12.9)	126 (11.4)	45 (20.4)	<.001	114 (11.2)	57 (18.4)	.003
0.5 (former smoker)	432 (32.5)	361 (32.6)	71 (32.1)		334 (32.8)	98 (31.7)	
1 (never smoker)	725 (54.6)	620 (56.0)	105 (47.5)		571 (56.0)	154 (49.8)	
Alcohol consumption							
0 (>1 medium drink per day)	132 (9.9)	276 (27.5)	102 (33.2)	.04	98 (9.5)	34 (11.0)	.18
0.5 (≤1 medium drink per day)	757 (56.5)	330 (32.9)	107 (34.9)		596 (57.9)	161 (51.9)	
1 (never consume alcohol)	451 (33.7)	396 (39.5)	98 (31.9)		336 (32.6)	115 (37.1)	
Lifestyle index (0-7)^h^							
Lowest tertile (≤3.0)	378 (28.9)	276 (27.5)	102 (33.2)	.04	298 (27.3)	80 (36.5)	.001
Middle tertile (3.5-4.0)	437 (33.4)	330 (32.9)	107 (34.9)		358 (32.8)	79 (36.1)	
Highest (healthiest) tertile (4.5-7.0)	494 (37.7)	396 (39.5)	98 (31.9)		434 (39.8)	60 (27.4)	

Abbreviations: BMI, body mass index (calculated as weight in kilograms divided by height in meters squared); GED, general educational development; MET, metabolic equivalent of task; MVPA, moderate or vigorous recreational physical activity; NA, not applicable; SPM, servings per month; SPW, servings per week.

^a^ Data are presented as number (percentage) of patients unless otherwise indicated.

^b^ Sample size may not sum to total because of missing data: less than 1.0% missingness for educational attainment (n = 6), race (n = 4), menopause (n = 14), lymph node (n = 2), *ERBB2* (n = 6), hormone receptor (n = 3), subtype (n = 8), BMI (n = 10), sugar-sweetened beverage (n = 4), and smoking (n = 12). No data were missing for age, physical activity, fruit and vegetable intake, red and processed meat intake, and alcohol.

^c^ *P* values reflect pooled *t* test for survival time, age, and BMI (continuous); otherwise, the χ^2^ test was used and values are rounded to the nearest 1000th place except where *P* < .001.

^d^ Other race includes women self-identifying as an unknown race other than African American/Black, American Indian, Asian, multiracial, Pacific Islander, or White.

^e^ Inactive patients engaged in no regular MVPA in the month before diagnosis; insufficiently active patients engaged in some regular MVPA but less than 7.5 MET h/wk, and patients meeting or exceeding the guidelines engaged in 7.5 MET h/wk or more.

^f^ Tertiles of intake for fruit and vegetable and red and processed meat consumption are categorized according to the mean number of medium-size servings per week during the month before diagnosis.

^g^ Tertiles of intake for sugar-sweetened beverage consumption are categorized according to the mean number of medium-size servings per month during the month before diagnosis.

^h^ The lifestyle index score is composed of 7 individual lifestyles reported in the month before diagnosis and ranges from 0 to 7, with 7 indicative of healthiest combined lifestyle and 0 the least healthy lifestyle.

**Figure 2.**
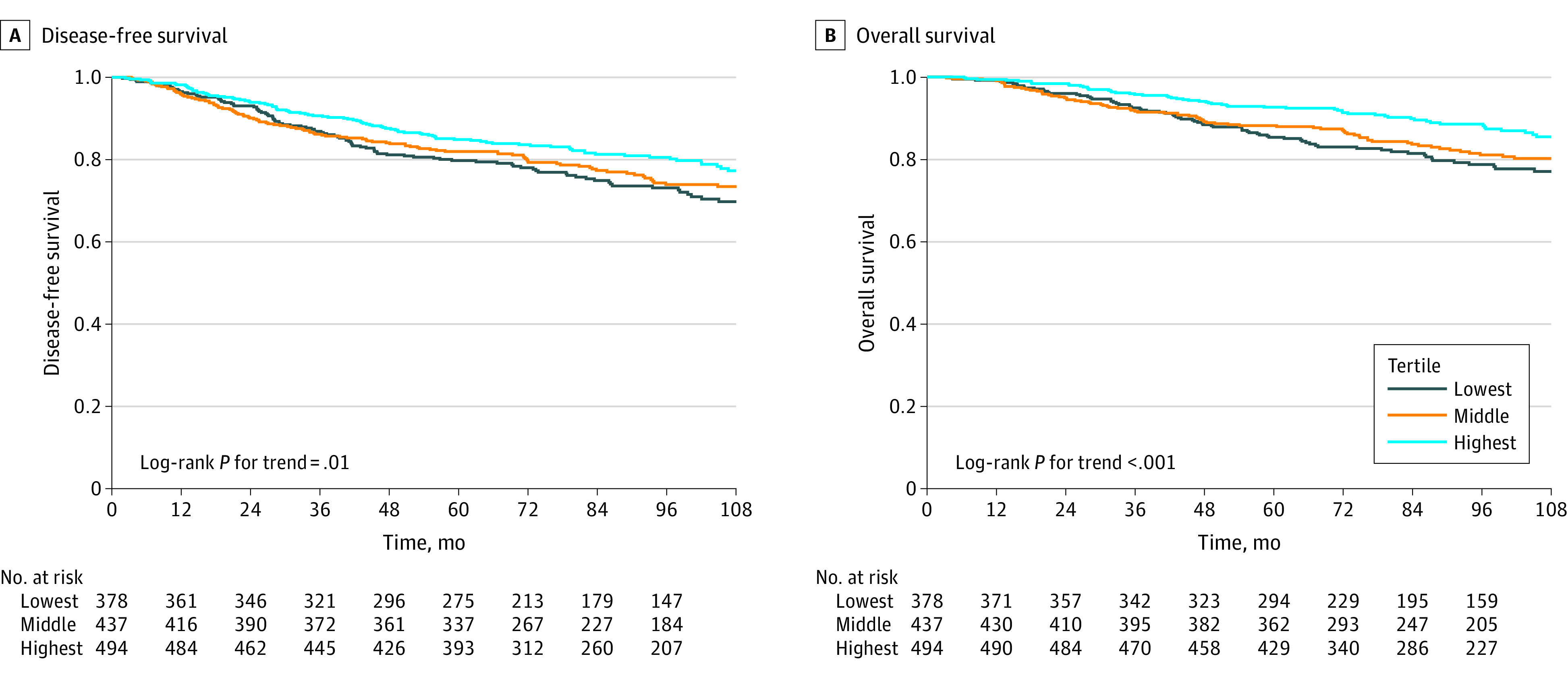
Disease-Free and Overall Survival Experience in the Diet, Exercise, Lifestyles, and Cancer Prognosis (DELCaP) Study According to the Lifestyle Index Score Kaplan-Meier plots representing associations of lifestyle index score tertiles at baseline with disease-free survival (A) and overall survival (B) in the DELCaP Study. The highest tertile reflects the strongest adherence to the American Institute for Cancer Research and American Cancer Society cancer prevention recommendations.

In univariable analyses assessing associations of participant characteristics at baseline with BC outcomes, age, menopause status, number of positive nodes, *ERBB2* and hormone receptor status, tumor subtype, PA, smoking status, alcohol consumption, and the LIS were significantly associated with disease recurrence (Table). Similarly, age, educational attainment, menopause status, number of positive nodes, *ERBB2* and hormone receptor status, tumor subtype, smoking status, and the LIS were significantly associated with mortality. In additional univariable analyses, the aggregated LIS was significantly associated with age, educational attainment, race, and number of positive nodes (eTable in Supplement 1). In Kaplan-Meier analyses, significant differences in disease-free (log-rank *P* = 0.01 for trend) and overall survival (log-rank *P*<.001 for trend) according to LIS tertiles were observed, with strongest adherence associated with longer survival (Figure 2).

**Figure 3.**
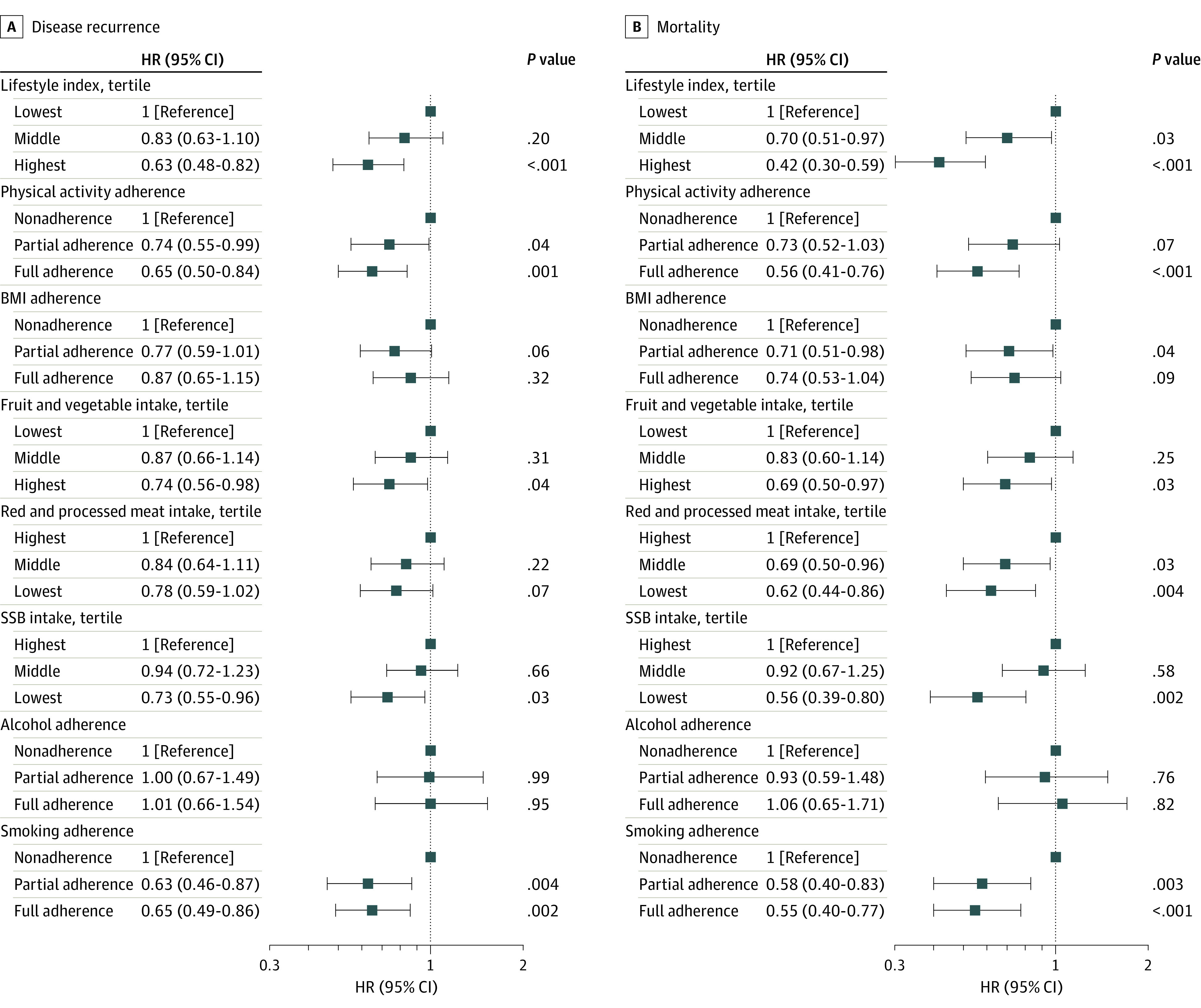
Time-Dependent Multivariable Associations of Lifestyle Adherence Scores With Disease Recurrence and Mortality in the Diet, Exercise, Lifestyles, and Cancer Prognosis (DELCaP) Study Forest plots depicting the hazard ratios (HRs) and 95% CIs representing the time-varying associations of the aggregate lifestyle index and individual lifestyle scores with disease recurrence (A) and mortality (B) in the DELCaP Study. For each lifestyle domain and the lifestyle index, the lowest level of adherence (ie, the least healthy behavior) served as the referent group. All multivariable models were adjusted for age at study enrollment, number of positive nodes, tumor subtype, and a stratification factor for treatment arm in Southwest Oncology Group S0221 trial. A, For disease recurrence, significant dose-dependent associations were observed for physical activity (*P* < .001 for trend), fruit and vegetable intake (*P* = .04 for trend), sugar-sweetened beverage (SSB) consumption (*P* = .03 for trend), and smoking status (*P* = .01 for trend). B, For mortality, significant dose-dependent associations were observed for physical activity (*P* < .001 for trend), body mass index (BMI) (*P* = .05 for trend), food and vegetable consumption (*P* = .03 for trend), red and processed meats (*P* = .003 for trend), SSB consumption (*P* = .002 for trend), and smoking (*P* = .002 for trend).

Forest plots representing time-varying multivariable associations of the aggregated LIS with BC outcomes are presented in Figure 3. Patients with the highest vs lowest LIS experienced significant reductions in recurrence (hazard ratio [HR], 0.63; 95% CI, 0.48-0.82). Although the association for the middle LIS tertile was not significant (HR, 0.83; 95% CI, 0.63-1.10), a significant dose-dependent association was observed (*P* < .001 for trend). Moreover, patients with an LIS in the middle and highest vs lowest tertile experienced significant reductions in mortality (HR, 0.70; 95% CI, 0.51-0.97 and HR, 0.42; 95% CI, 0.30-0.59, respectively; *P* < .001 for trend).

In time-varying analyses for each lifestyle, partial and full adherence to the PA and smoking recommendations and full adherence to fruit and vegetable and sugar-sweetened beverage recommendations were associated with reduced disease recurrence; no statistically significant associations were observed for BMI, red and processed meats, or alcohol consumption (Figure 3A). Additionally, full adherence to PA, smoking, fruit and vegetable, and sugar-sweetened beverage recommendations and partial and full adherence to red and processed meat recommendation were associated with significant reductions in mortality (Figure 3B). For BMI, maintaining a normal weight was not significantly associated with mortality, but overweight was associated with significantly reduced mortality (HR, 0.71; 95% CI, 0.51-0.98). No statistically significant association was observed between alcohol consumption and mortality.

Multivariable associations of the LIS with BC outcomes at each time point (Q1-Q4) are presented in eFigures 2 through 5 in Supplement 1, respectively. Highest vs lowest LIS was associated with reduced recurrence and mortality at Q1 (eFigure 2 in Supplement 1), reduced mortality at Q3 (eFigure 4B in Supplement 1), and reduced recurrence at Q4 (eFigure 5A in Supplement 1). Adherence to the smoking, PA, and red and processed meat recommendations were most consistently associated with outcomes at each time point (eFigures 2-5 in Supplement 1).

**Figure 4.**
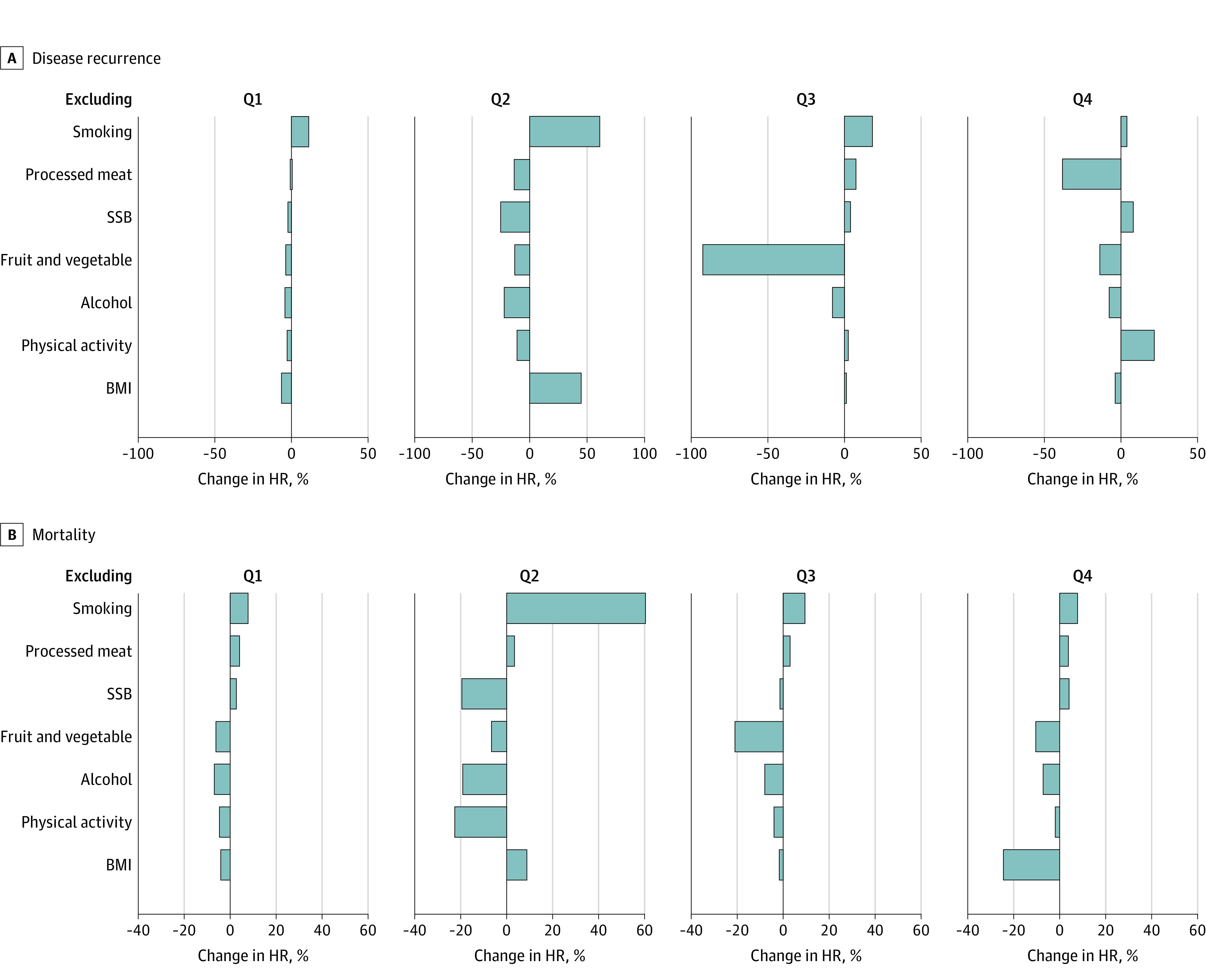
A Summary of Leave-Out Analyses Depicting the Robustness of the Lifestyle Index Score to the Influence of Any Individual Lifestyle Domain Leave-out analyses depict the percent change in hazard ratios (HRs) representing the association of the aggregate lifestyle index score with hazards of disease recurrence (A) and hazards of mortality (B) in the Diet, Exercise, Lifestyles, and Cancer Prognosis (DELCaP) Study at 4 time points (Q1-Q4). Questionnaire 1 (Q1) represents lifestyles before diagnosis, Q2 represents lifestyles during treatment, Q3 represents lifestyles 1 year after enrollment (6 months after treatment completion), and Q4 represents lifestyles 2 years after enrollment. Bars to the right of the vertical lines represents a positive percent change in effect, indicating that the HR representing the association of the lifestyle index with survival was attenuated when that factor was excluded (ie, the HR increased). A negative percent change indicates the HR decreased (ie, the association was strengthened) when the factor was excluded. Thus, the factor with the largest positive percent increase was the most important contributor to the association of the lifestyle index with the outcome at the respective time point. BMI indicates body mass index; SSB, sugar-sweetened beverage.

Leave-out analyses for disease recurrence revealed that adherence to the smoking recommendation at Q1 to Q3 and the PA recommendation at Q4 yielded the highest positive percent change in estimate when removed from the LIS (Figure 4A). However, for mortality, smoking status was the most important contributor to the LIS-mortality association at all 4 time points (Figure 4B).

In sensitivity analyses designed to assess the possibility of selection bias, differences in the 5-year survival of patients enrolled in DELCaP (88.0%) with patients enrolled in S0221 (89.0%) were negligible, with event rates of 0.026 and 0.023, respectively.^20,21^ Minimal differences in successive response rates for Q1 to Q4 according to lifestyles were also noted. For example, patients with highest vs lowest LIS at Q1 were only slightly more likely to respond at Q2 (5.4%) and Q3 (3.9%), but negligible differences were observed at Q4 (0.4%).

Next, in quantitative analyses that assessed the role of unmeasured confounding, the E-values were 2.10 for disease recurrence and 3.03 for mortality.^31^ Last, we found no evidence that the exposure-outcome association was confounded or modified by menopause status (*P* = .82 for interaction), race (*P* = .84 for interaction), educational attainment (*P* = .72 for interaction), or tumor subtype (*P* = .65 for interaction). For example, in subgroup analyses according to tumor subtype (eFigure 6 in Supplement 1), significant decreases in mortality were consistently observed for highest vs lowest LIS among patients with hormone receptor–positive, *ERBB2*-negative tumors (HR, 0.45; 95% CI, 0.26-0.80), triple-negative BC (HR, 0.47; 95% CI, 0.29-0.76), and *ERBB2*-positive tumors (HR, 0.25; 95% CI, 0.08-0.76).

## Discussion

In this prospective cohort study of adherence to cancer prevention guidelines before, during, and after treatment for high-risk BC, strongest adherence to cancer prevention lifestyle recommendations was associated with a 58% reduction in mortality and a 37% reduction in disease recurrence. Associations were not modified by educational attainment, self-identified race or ethnicity, or menopause status, and significant reductions in recurrence and mortality were consistently observed even among patients diagnosed with more aggressive BC subtypes.

Although the putative influences of diet, exercise, and smoking on the cellular processes underpinning the progression of BC have been extensively reviewed,^1,2,19^ to our knowledge, this is the first report showing that lifestyles before, during, and after chemotherapy were associated with improved outcomes in patients with high-risk BC. Although no prior reports have described associations of an aggregated LIS from multiple time points with high-risk BC outcomes, our findings coincide with previous reports showing that healthier lifestyle scores are associated with better survival in patients diagnosed with a variety of tumors.^19,32,33,34,35,36,37,38,39,40^

In examining the role of individual lifestyles, strongest adherence to recommendations for smoking, PA, fruit and vegetable intake, and sugar-sweetened beverage consumption were associated with significant reductions in recurrence and mortality. However, never smoking and meeting or exceeding the PA guidelines yielded the most consistent and robust associations with outcomes, with each factor associated with a 44% to 45% reduced hazard of mortality and a 35% reduced hazard of recurrence. These findings were confirmed in leave-out analyses, showing PA and smoking yielded the largest positive percent change in effect when removed from the LIS at each time point.

Conversely, strongest adherence to the alcohol and BMI recommendations was not significantly associated with improved outcomes, but overweight was associated with significantly improved survival. These findings are not entirely unexpected, because conflicting evidence and competing hypotheses regarding associations of alcohol and BMI with survival exist and associations may not be linear.^13,15,41,42,43,44,45,46,47,48,49,50^ For example, overweight is often associated with improved BC survival in the extant literature (ie, an overweight paradox).^48,49,50^ Although viable biological pathways have been proposed, methodologic issues, such as reliance on BMI as a proxy for adiposity or collider bias, may underly observed survival advantages among patients with overweight herein and in the literature.^48,49,50,51^

### Strengths and Limitations

Important strengths of our study include the large, well-characterized population of patients with BC, repeated lifestyle assessments using validated questionnaires, and the ability to control for treatment regimens. Importantly, incorporation of exposure data collected at multiple time points likely offset biases that could ensue from relying solely on prediagnosis or postdiagnosis exposures, which could be influenced by disease- and treatment-related symptoms.

The DELCaP Study included patients with BC enrolled in a clinical trial; thus, these findings may not be generalizable to more diverse clinical populations. Additionally, although we assessed the influence of measured and unmeasured confounders, we cannot rule out the possibility that residual confounding influenced our results.^21^ We also cannot account for unmeasured factors (ie, quality of life after Q4) that may mediate the observed association between lifestyles and BC outcomes. However, the calculated E-values of 2.10 for disease recurrence and 3.03 for mortality reflect the minimum magnitude of association needed for unmeasured confounder(s) to have with both the exposure and outcome to explain away observed associations.^31^ Given that HRs of 2- and 3-fold are not commonly observed in biomedical literature, an unmeasured variable that affects both the exposure and the outcome of interest by this magnitude would be even less common.^31^

Moreover, because BC-specific survival was not tracked in S0221, the primary outcome is all-cause mortality. Consequently, we cannot account for comorbidities that may have developed after treatment completion, such as cardiovascular disease, a major competing cause of death among older patients with BC.^21,52^ However, because patients with comorbidities, poor performance status, or a subnormal ejection fraction were excluded from S0221, competing causes of cardiovascular mortality may have been less likely to contribute to events in this study population.^21^

We cannot rule out the possibility that selection biases (ie, healthy survivor bias) influenced our findings. However, in a series of sensitivity analyses, we found that differences in survival among patients enrolled in S0221 vs DELCaP were negligible. Moreover, there was no convincing evidence that patients with less healthy lifestyles were more likely to be lost to follow-up. Collectively, these analyses lessened our concern that a healthy survivor bias was at play.

## Conclusion

Strongest collective adherence to cancer prevention recommendations before, during, and after treatment was associated with significant reductions in disease recurrence and mortality among patients with high-risk BC in the DELCaP Study. Strongest adherence to recommendations for smoking, PA, fruit and vegetable intake, and sugar-sweetened beverage consumption was most consistently associated with improved outcomes. Importantly, significant survival advantages were consistently observed in patients diagnosed with more aggressive BC subtypes.

Although strong evidence supporting the incorporation of smoking cessation and PA interventions during survivorship exists, additional confirmatory studies are needed to solidify the survival benefits of dietary and weight loss interventions.^53,54,55^ Whereas expensive and potent therapeutics provide the foundation for BC treatment, lifestyle interventions could be a safe, inexpensive, and feasible ancillary strategy for delaying and preventing recurrence and death from the most common cancer in the world. Such developments could be especially impactful for patients diagnosed with more aggressive tumors that do not respond well to current therapies.

## Notes

Supplement 1. **eMethods.** Supplemental Methods **eTable.** Epidemiological and Clinical Characteristics of the DELCaP Study Population According to the Aggregate Lifestyle Index Score **eFigure 1.** Directed Acyclic Graphs **eFigure 2.** Hazard Ratios (HR) and 95% Confidence Intervals (CI) Representing Associations of the Lifestyle Index Score (LIS) and Individual Lifestyles Before Diagnosis (Q1) With (A) Disease Recurrence and (B) Mortality in the DELCaP Study **eFigure 3.** Hazard Ratios (HR) and 95% Confidence Intervals (CI) Representing Associations of the Lifestyle Index Score (LIS) and Adherence to Individual Lifestyle Recommendations During Treatment (Q2) With (A) Disease Recurrence and (B) All-Cause Mortality in the DELCaP Study **eFigure 4.** Hazard Ratios (HR) and 95% Confidence Intervals (CI) Representing Associations of the Lifestyle Index Score and Adherence to Individual Lifestyle Recommendations at One-Year Follow-Up (Q3) With (A) Disease Recurrence and (B) All-Cause Mortality in the DELCaP Study **eFigure 5.** Hazard Ratios (HR) and 95% Confidence Intervals (CI) Representing Associations of the Lifestyle Index Score and Adherence to Individual Lifestyle Recommendations at Two-Year Follow-up (Q4) With (A) Disease Recurrence and (B) All-Cause Mortality in the DELCaP Study **eFigure 6.** Hazard Ratios (HR) and 95% Confidence Intervals (CI) Representing Associations of the Aggregate Lifestyle Index Score With (A) Disease Recurrence and (B) All-Cause Mortality According to Tumor Subtype in the DELCaP Study
eReferences

Supplement 2.
Data Sharing Statement
