# Supplementary material for: Adherence to Cancer Prevention Lifestyle Recommendations Before, During, and 2 Years After Treatment for High-risk Breast Cancer
Source: JAMA Netw Open. 2023 May 4;6(5):e2311673. doi: 10.1001/jamanetworkopen.2023.11673 (PMC10160875; doi:10.1001/jamanetworkopen.2023.11673)
Supplement: Supplement 1. — eMethods. Supplemental Methods eTable. Epidemiological and Clinical Characteristics of the DELCaP Study Population According to the Aggregate Lifestyle Index Score eFigure 1. Directed Acyclic Graphs eFigure 2. Hazard Ratios (HR) and 95% Confidence Intervals (CI) Representing Associations of the Lifestyle Index Score (LIS) and Individual Lifestyles Before Diagnosis (Q1) With (A) Disease Recurrence and (B) Mortality in the DELCaP Study eFigure 3. Hazard Ratios (HR) and 95% Confidence Intervals (CI) Representing Associations of the Lifestyle Index Score (LIS) and Adherence to Individual Lifestyle Recommendations During Treatment (Q2) With (A) Disease Recurrence and (B) All-Cause Mortality in the DELCaP Study eFigure 4. Hazard Ratios (HR) and 95% Confidence Intervals (CI) Representing Associations of the Lifestyle Index Score and Adherence to Individual Lifestyle Recommendations at One-Year Follow-Up (Q3) With (A) Disease Recurrence and (B) All-Cause Mortality in the DELCaP Study eFigure 5. Hazard Ratios (HR) and 95% Confidence Intervals (CI) Representing Associations of the Lifestyle Index Score and Adherence to Individual Lifestyle Recommendations at Two-Year Follow-up (Q4) With (A) Disease Recurrence and (B) All-Cause Mortality in the DELCaP Study eFigure 6. Hazard Ratios (HR) and 95% Confidence Intervals (CI) Representing Associations of the Aggregate Lifestyle Index Score With (A) Disease Recurrence and (B) All-Cause Mortality According to Tumor Subtype in the DELCaP Study eReferences [file jamanetwopen-e2311673-s001.pdf]

Supplementary Online Content

Cannioto RA, Attwood KM, Davis EW, et al. Adherence to cancer prevention lifestyle recommendations before, during, and 2 years after treatment for high-risk breast cancer. *JAMA Netw Open*. 2023;6(5):e2311673. doi:10.1001/jamanetworkopen.2023.11673

eMethods. Supplemental Methods

eTable. Epidemiological and Clinical Characteristics of the DELCaP Study Population According to the Aggregate Lifestyle Index Score

eFigure 1. Directed Acyclic Graphs

eFigure 2. Hazard Ratios (HR) and 95% Confidence Intervals (CI) Representing Associations of the Lifestyle Index Score (LIS) and Individual Lifestyles Before Diagnosis (Q1) With (A) Disease Recurrence and (B) Mortality in the DELCaP Study

eFigure 3. Hazard Ratios (HR) and 95% Confidence Intervals (CI) Representing Associations of the Lifestyle Index Score (LIS) and Adherence to Individual Lifestyle Recommendations During Treatment (Q2) With (A) Disease Recurrence and (B) All-Cause Mortality in the DELCaP Study

eFigure 4. Hazard Ratios (HR) and 95% Confidence Intervals (CI) Representing Associations of the Lifestyle Index Score and Adherence to Individual Lifestyle Recommendations at One-Year Follow-Up (Q3) With (A) Disease Recurrence and (B) All-Cause Mortality in the DELCaP Study

eFigure 5. Hazard Ratios (HR) and 95% Confidence Intervals (CI) Representing Associations of the Lifestyle Index Score and Adherence to Individual Lifestyle Recommendations at Two-Year Follow-up (Q4) With (A) Disease Recurrence and (B) All-Cause Mortality in the DELCaP Study

eFigure 6. Hazard Ratios (HR) and 95% Confidence Intervals (CI) Representing Associations of the Aggregate Lifestyle Index Score With (A) Disease Recurrence and (B) All-Cause Mortality According to Tumor Subtype in the DELCaP Study

eReferences

This supplementary material has been provided by the authors to give readers additional information about their work.

© 2023 Cannioto RA et al. *JAMA Network Open.*

eMethods. Supplemental Methods

Lifestyle Assessment and Standardized Adherence Scoring

Recreational Physical Activity (RPA) Assessment. The DELCaP questionnaire assessed mode, frequency and duration of RPA and was adapted from the Lifetime Physical Activity Questionnaire, a self-administered survey with established reliability among adult women[1]. Total MET minutes and hours were calculated for each person at each time based upon corresponding codes and MET values published in the Physical Activity Compendium[2]. Activities were included in the analysis if performed at least once per week throughout the exposure window assessed and if the compendium MET value was at least 3.0 or higher[2-4].

For the Lifestyle Index Score (LIS), patients who achieved, on average, ≥7.5 MET hours per week (i.e., the equivalent of 150 minutes of moderate-intensity RPA per/week)[5] were classified as adhering to the Physical Activity Guidelines and the ACS/AICR recommendation (1); patients who were regularly exercising but achieving insufficient activity (<7.5 MET hours/week) were classified as partially adherent (.5) and patients who did not engage in any regular exercise were assigned a score of zero.

It is important to note that in our analyses we rated non-adherence and partial adherence to the PA Guidelines differently than previously proposed. Standardization guidelines suggest patients engaging in up to 74 minutes of moderate to vigorous PA per week be classified as non-adherent. However, evidence from our group and others shows that low volumes of regular, weekly activity, in comparison to inactivity, are associated with significant reductions in all-cause and cancer-specific mortality[6,7]. Thus, we classified patients who were inactive as non-adherent and patients who were insufficiently active (i.e., regularly active but less than 7.5 MET hrs/week) as partially adherent.

Body Mass Index Assessment and Parameterization. Height and weight were queried upon study enrollment and current weight was queried at each questionnaire administration. BMI was calculated as weight in kilograms divided by height in meters squared and was classified according to the World Health Organization guidelines as: underweight (BMI<18.5 kg/m^2); normal weight (BMI=18.5-24.9 kg/m^2); overweight (BMI=25.0-29.9 kg/m^2); and obese (BMI= ≥30 kg/m^2) [8]. For the lifestyle index score, patients classified as normal weight were scored as adherent to the recommendation (1); patients who were overweight were considered partially adherent (.5) and patients who were underweight or obese were scored as non-adherent (0).

Food Frequency Questionnaire. A 110-item food frequency questionnaire (FFQ) was included in the DELCaP questionnaire and was used for determining adherence to the ‘Fruit and Vegetables’, ‘Processed Meats’, ‘Sugar Sweetened Beverages’ and ‘alcohol consumption’ recommendations. The FFQ was adapted from the validated questionnaire used in the Vitamins and Lifestyle (VitaL) study[9]. At each timepoint, participants indicated the frequency of consumption (never, 1 per month, 2–3 per month, 1 per week, 2 per week, 3–4 per week, 5–6 per week, 1 per day, and 2+ per day) and the portion size (small, medium, large) for each food. Beverage consumption frequency included never, < 1 per month, 1–3 per month, 1 per week, 2–4 per week, 5–6 per week, 1 per day, 2–3 per day, 4–5 per day, and 6+ per day with small, medium, and large serving size options. Total consumption for each dietary recommendation was computed by multiplying frequency per month by portion size using a value of 0.5 for small, 1 for medium, and 1.5 for large portions.

Fruit and Vegetable Consumption. The FFQ surveyed consumption of a variety of fruits, vegetables and legumes. For each fruit, vegetable, and legume group, patients indicated the frequency of consumption (never, 1 per month, 2–3 per month, 1 per week, 2 per week, 3–4 per week, 5–6 per week, 1 per day, and 2+ per day) and the portion size (small, medium, large). Total consumption for each item was computed as described above and were summed and divided into tertiles. The lowest tertile of intake was assigned a zero; middle tertile a .5 for partial adherence and the highest tertile a 1 for strongest adherence to the recommendation.

Red and Processed Meats. The FFQ surveyed a variety of red and processed meats (e.g., bacon, breakfast sausage, hot dogs, sausage, lunch meats, beef, pork, ham, lamb). For each item, patients indicated the frequency of consumption (never, 1 per month, 2–3 per month, 1 per week, 2 per week, 3–4 per week, 5–6 per week, 1 per day, and 2+ per day) and the portion size (small, medium, large). Total consumption for each item was computed and divided into tertiles. The lowest tertile of intake was assigned a 1 for strongest adherence; the middle tertile was assigned a .5 for partial adherence, and the highest tertile a 0 for non-adherence to the recommendation.

© 2023 Cannioto RA et al. *JAMA Network Open.*

Sugar Sweetened Beverages. The FFQ surveyed sweetened beverages including regular soft drinks, fruit drinks, juice cocktails, hot chocolate and other sweetened hot beverages (latte, mocha) and meal replacement drinks. Total consumption for was computed by multiplying frequency (never, < 1 per month, 1–3 per month, 1 per week, 2–4 per week, 5–6 per week, 1 per day, 2–3 per day, 4–5 per day, and 6+ per day) by portion size using a value of 0.5 for small, 1 for medium, and 1.5 for large portions. Total consumption per month was computed and divided into tertiles. The lowest tertile of intake was assigned a 1 for strongest of adherence; the middle tertile was assigned a .5 for partial adherence, and the highest tertile a 0 for non-adherence to the recommendation.

Alcohol. The AICR and ACS recommends avoiding or limiting alcohol consumption to ≤1 drink per day. For alcohol intake, the DELCaP questionnaire queried the type of alcohol consumed (beer, wine, liquor and mixed drinks), the size of portion consumed (small, medium, large) and the frequency per month, week or day. A medium serving size equates to one drink and includes 12 ounces of beer, 4 ounces of wine or 1.5 ounces of liquor. Small serving size was assigned a value of .5 drinks and large serving size was assigned a value of 1.5 drinks. Patients indicating that they never drank were assigned a score of 1 (full adherence). Patients reporting ≤ one medium drink per day were assigned a half point (.5) for partial adherence; and patients reporting ≥ one large drink per day were assigned a zero.

Smoking. For the lifestyle index, current smokers were classified as non-adherent and assigned a score of zero. Former smokers were classified as partially adherent and assigned a score of .5. Finally, never smokers were defined as smoking less than 100 cigarettes in their lifetime and were classified as adherent and assigned a score of 1.

Lifestyle Scoring. In compliance with standardized published scoring guidance, for each lifestyle, a score of one point represents the healthiest behavior or strongest level of adherence, a half point for partial adherence, and a zero for non-adherence or the least healthy behavior. For PA, BMI, smoking and alcohol, complete adherence to prevention recommendations was assigned a one, partial adherence a half-point, and non-adherence a score of zero. For sugar-sweetened beverage and red and processed meat consumption, the lowest tertile of consumption was assigned a one, middle tertile a half point, and the highest tertile a zero. Conversely, for fruit and vegetable intake, the highest tertile of consumption was assigned a score of one, middle tertile a half point and the lowest tertile a zero. To create the composite Lifestyle Index Score, individual adherence scores were combined, with total scores ranging between 0 and 7. Finally, the composite Lifestyle Index Scores at each timepoint and the aggregate score were categorized into tertiles, with the highest tertile indicative of the most adherent, healthiest lifestyle, and the lowest tertile indicative of the least healthy lifestyle.

Assessment of Confounding

We employed several well-established conceptual and empirical methods for identifying confounding pathways. First, we identified factors for consideration if they met the conceptual criteria and definition of confounding including: (i) the variable is known to be associated with the exposure but not affected by or caused by the exposure; (ii) the variable is known to be a cause or surrogate cause of the outcome (i.e., prognostic factor or surrogate prognostic factor); and (iii) the variable is not affected by the exposure or in the causal pathway between the exposure or the outcome[10]. For identifying conceptual confounders, we relied on the extant literature and Directed Acyclic Graphs (DAGs) to assist in distinguishing classical confounders from pathway variables or colliders (**eFigure 1**).

Second, we used data-driven approaches to identify measured covariates that were statistically associated with both the exposure and the outcome of interest. To accomplish this, we examined univariable statistical associations of putative confounders with BC outcomes (**Table 1**) and the aggregate LIS (**eTable**) using p<0.20 as a cutoff for consideration[11]. Next, in multivariable analyses, we used the 10% change-in-estimate guideline[11] and stepwise regression to examine the influence of each putative confounder in multivariable models.

Based on DAGs (**eFigure 1)**, age, self-identified race, ethnicity, education, menopause status, and other unmeasured factors (i.e., lifestyles in the years prior to study enrollment, SES, built environment, etc.) were identified in the conceptual model of confounding for both disease recurrence (**eFigure 1a**) and all-cause mortality (**eFigure 1b**). However, for the LIS-recurrence association, post-Q4 unmeasured factors (i.e. factors temporally

© 2023 Cannioto RA et al. *JAMA Network Open.*

occurring after the primary exposure of interest) including lifestyles, performance status, newly developed comorbidities or newly developed treatment toxicities could each be affected by the primary exposure and the primary outcome (a breast cancer recurrence), thus collider bias could ensue with adjustment for these factors[12]. Conversely, in the LIS-mortality association (**eFigure 1b**), we show that unmeasured factors occurring after Q4 (i.e., lifestyles after Q4, performance status, QoL, newly developed comorbidities or newly developed treatment toxicities) could be mediators because they would be downstream of the primary exposure of interest, in the causal pathway, and associated with all-cause mortality.

Based upon univariable analyses, education, race, and the number of positive nodes were associated with both the exposure (**eTable**) and outcomes (**Table 1**) of interest, while menopause status and BC subtype were only associated with BC outcomes. In multivariable analyses, adjustment for race, ethnicity, education and menopause status did not change estimates of association between the LIS and BC outcomes and these factors were not statistically significant factors in stepwise regression. For example, the association of the highest versus lower LIS with disease recurrence was (HR=0.63, 95% CI: 0.48, 0.82); when education and race were added to the model, the association was (HR=0.63, 95% CI: 0.48-0.84). Likewise, for the association of highest versus lowest LIS with mortality, we observed (HR=0.42, 95% CI:0.30-0.59); when education and race were added to the model, the association was (HR=0.43, 95% CI:0.30-0.61). Additionally, there was no evidence of effect modification or statistical interaction according to *LIS*race* (p-for-interaction = 0.84), *LIS*education* (p-for-interaction=0.72), *LIS*menopause status* (p-for-interaction=0.82) or *LIS*BC subtype* (p-for-interaction=0.65). Conversely, the number of positive nodes, HER2 status, and ER/PgR status were statistically significant factors in multivariable models assessing associations of the LIS with BC outcomes. Thus, fully adjusted multivariable models included age at study enrollment, number of positive nodes, HER2 and ER/PgR status, and a stratification factor for treatment arm.

Finally, to quantitatively assess the potential role of unmeasured confounding, we calculated the E-value. The E-value reflects the minimum association of unmeasured confounder(s) needed with both the exposure and outcome to explain away associations of the LIS with BC outcomes[13]. The E-values representing associations of highest versus lowest LIS with disease recurrence and mortality were 2.10 and 3.03, respectively. Given that HRs of 2- and 3-fold are not common in biomedical research, an unmeasured variable that affects both the exposure and the outcome of interest by this magnitude would be even less common[13].

Methods of Multivariable Modeling

As described in the main manuscript, we used two methods to investigate multivariable exposure-outcome associations. In primary multivariable analyses, time-dependent (i.e., time-varying) Cox models were used to assess associations of the aggregated LIS with BC outcomes. The aggregated LIS comprises seven lifestyle factors over four timepoints. In these models, the LIS or individual lifestyle factors are treated as time-dependent covariates, which accounts for the changing behavior of individuals over time. Moreover, time-dependent models assess short-term effects (i.e., HRs represent weighted averages of the association between the aggregate LIS and the outcome for each exposure window prior to an event)[14,15].

In secondary multivariable analyses, we used standard Cox models to assess associations of the LIS with BC outcomes at Q1. For Q2 through Q4, we used landmark analyses to investigate associations of the LIS with BC outcomes at each timepoint. The advantage of landmark analyses is in reducing the possibility of immortal time bias. However, as the landmark time became shorter with each successive questionnaire and data points were lost, these analyses provide an incomplete representation of the exposure-outcome association[15]. Additionally, unlike time-varying models, standard models assess long-term effects from one time point (i.e., time of study enrollment for Q1 or landmark time for Q2-Q4).

© 2023 Cannioto RA et al. *JAMA Network Open.*

eTable. Epidemiological and Clinical Characteristics of the DELCaP Study Population According to the Aggregate Lifestyle Index Score

Patient Characteristics	Low LIS[1] (N=652, 48.7%)	High LIS[1] (N=688, 51.3%)	p-value
**Age at Enrollment**	50.8 (±9.5)	51.8 (±10.3)	0.07
**Educational Attainment**			
No High School Diploma	51 (54.8%)	42 (45.2%)	<0.001
High School Graduate/GED	177 (61.7%)	110 (38.3%)	
Some College	257 (53.1%)	227 (46.9%)	
College Graduate	103 (36.0%)	183 (64.0%)	
Advanced Degree	60 (32.6%)	124 (67.4%)	
**Race**			
African American/Black	63 (67.0%)	31 (33.0%)	<0.001
American Indian	8 (61.5%)	5 (38.5%)	
Asian	6 (14.0%)	37 (86.1%)	
Multiracial	21 (46.7%)	24 (53.3%)	
Other[2]	8 (44.4%)	10 (55.6%)	
Pacific Islander	4 (80.0%)	1 (20.0%)	
White	540 (48.3%)	578 (51.7%)	
**Ethnicity**			
Hispanic	36 (55.4%)	29 (44.6%)	0.27
Non-Hispanic	616 (48.3%)	659 (51.7%)	
**Menopause Status**			
Pre-Menopausal	305 (48.4%)	325 (51.6%)	0.92
Post-Menopausal	339 (48.7%)	357 (51.3%)	
**Lymph Node Classification**			
Node Negative	193 (55.3%)	156 (44.7%)	0.009
1-3 Positive Nodes	242 (48.2%)	260 (51.8%)	
4+ Positive Nodes	217 (44.6%)	270 (55.4%)	
**HER-2 Status**			
HER2-Negative	512 (48.7%)	541 (51.3%)	0.92
HER2-Positive	135 (48.4%)	144 (51.6%)	
**Hormone Receptor Status**			
HR-Negative	236 (50.9%)	228 (49.1%)	0.26
HR-Positive	416 (47.65%)	457 (52.4%)	
**Tumor Subtype**			
HER2+	135 (48.4%)	144 (51.6%)	0.64
HR+; HER2-	336 (47.8%)	367 (52.2%)	
Triple Negative (HR-; HER2-)	178 (50.9%)	172 (49.1%)	

[1]The aggregate LIS is comprised of 7 lifestyles over four timepoints (Q1 through Q4) categorized at the median. Low LIS comprises the lower 50th percentile (lower adherence); High LIS comprises the top 50th percentile (stronger adherence/healthier lifestyles).

[2]Other race includes women self-identifying as an unknown race other than African American/Black, American Indian, Asian, Multiracial, Pacific Islander, or White

© 2023 Cannioto RA et al. *JAMA Network Open.*

eFigure 1. Directed Acyclic Graphs

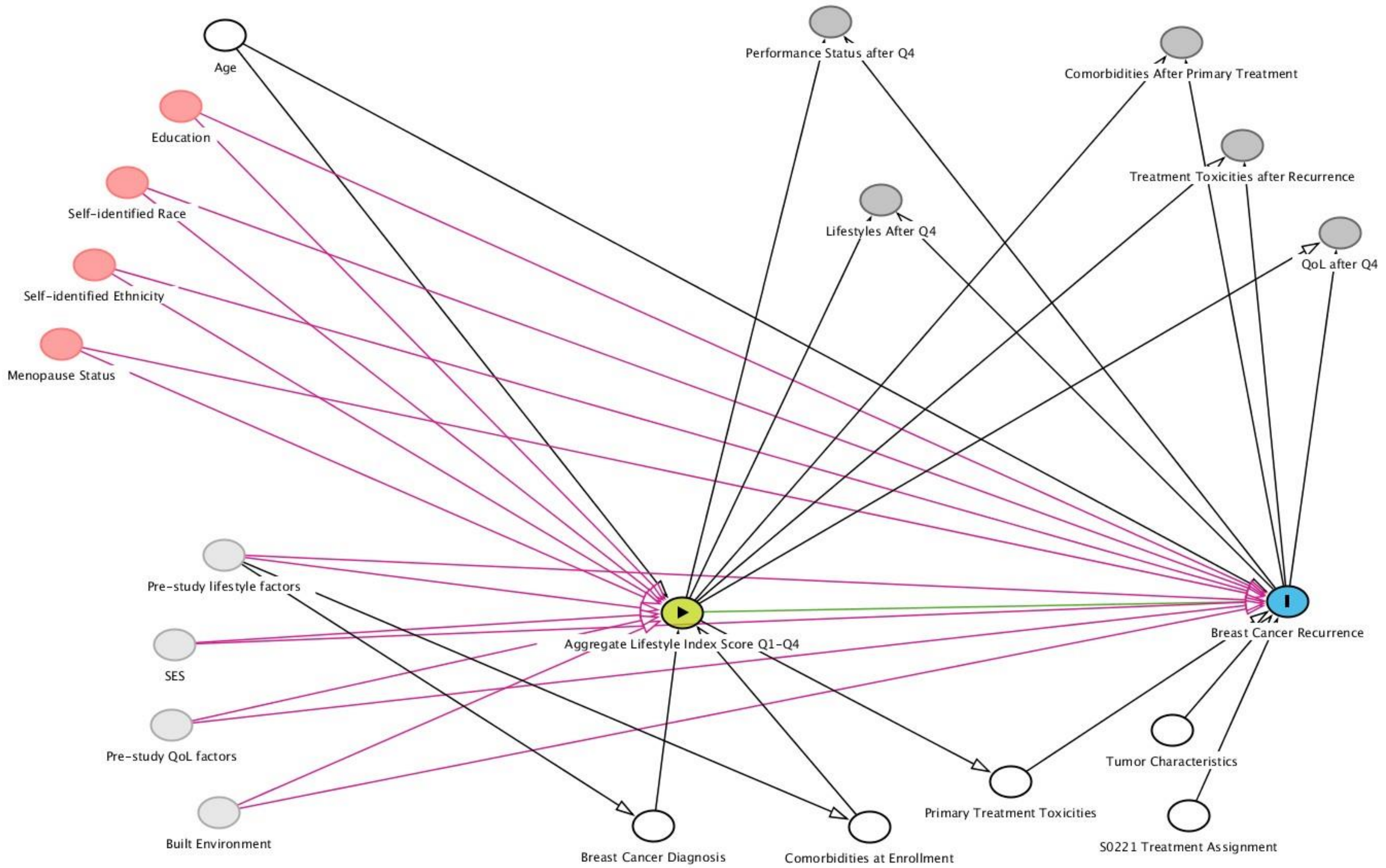

eFigure 1A. Directed Acyclic Graph Identifying Putative Classical Confounders and Colliders for Investigating the Association of the Aggregate Lifestyle Index Score at Q1 Through Q4 With Breast Cancer Recurrence

Pathways denoted by the green arrow represents the causal pathway between the primary analytic exposure and outcome of interest. Pathways denoted pink arrows represent classical confounding pathways; these factors influence the exposure and the outcome but are not in the causal pathway (i.e., they are not affected by the primary exposure of interest). Pink circles are measured confounders; light grey circles are unmeasured confounders for which we estimate possible influence using the E-value. White circles are factors which were controlled for by study design or through adjustment in multivariable models. Dark shaded grey variables are potential colliders because they are affected by both the primary exposure of interest and the primary outcome of interest (two causal pathways collide with adjustment for these factors). DAG created at daggity.net.

© 2023 Cannioto RA et al. *JAMA Network Open.*

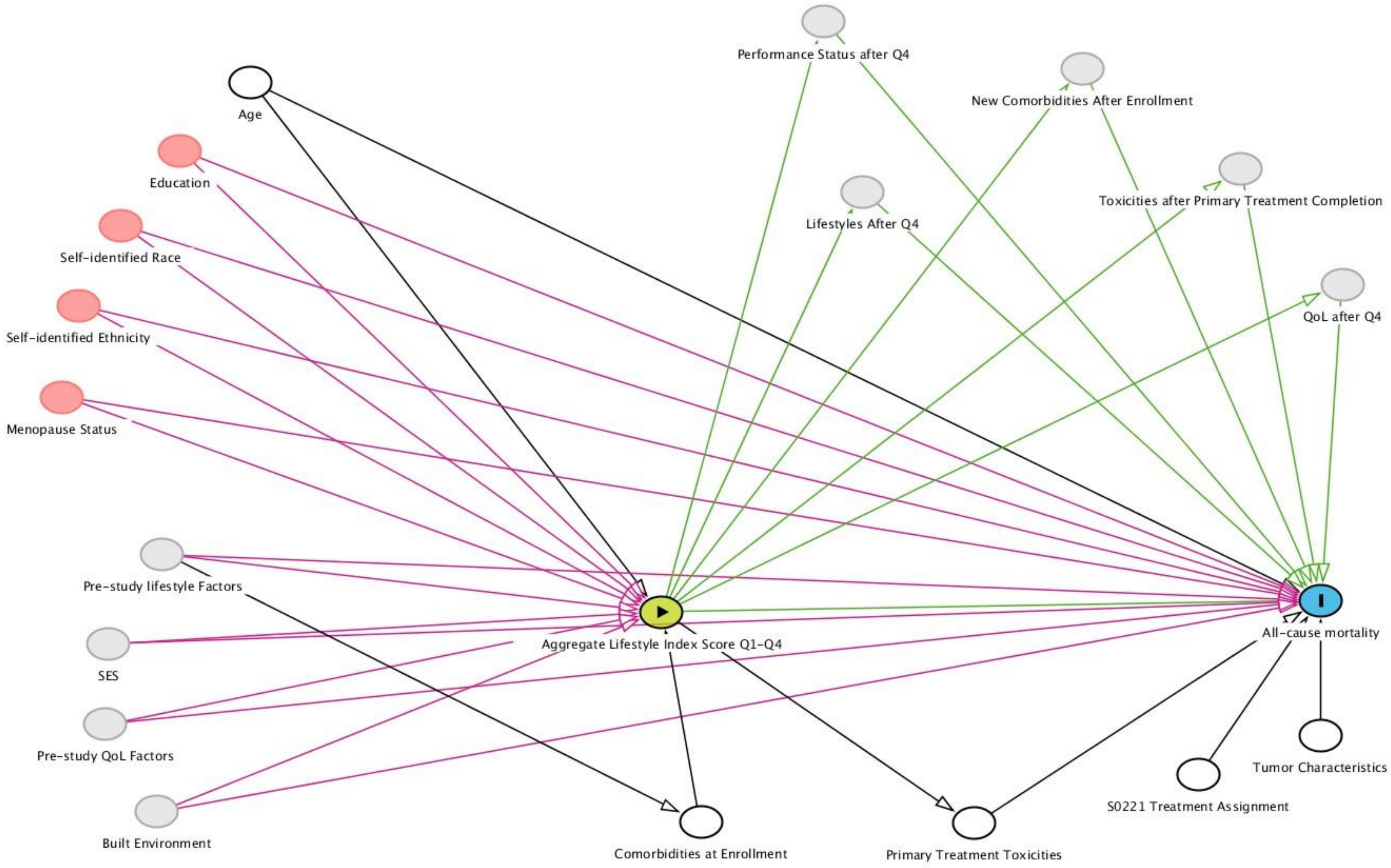

eFigure 1B. Directed Acyclic Graph Identifying Putative Classical Confounders and Mediating Variables in the Association of the Aggregate Lifestyle Index Score With All-Cause Mortality in the DELCaP Study

Pathways denoted by green arrows represent direct and indirect causal pathways between the primary analytic exposure and outcome of interest. Pathways denoted by pink arrows represent classical confounding pathways; these factors influence the exposure and the outcome but are not in the causal pathway (i.e., they are not affected by the primary exposure of interest). Pink circles are measured confounders; light grey circles connected with pink pathways are unmeasured confounders for which we estimate possible influence using the E-value. Light grey circles connected to green pathways are unmeasured factors that could potentially mediate the association between the aggregate LIS and all-cause mortality. White circles are factors that were controlled for by study design or through adjustment in multivariable models. DAG created at daggity.net.

© 2023 Cannioto RA et al. *JAMA Network Open.*

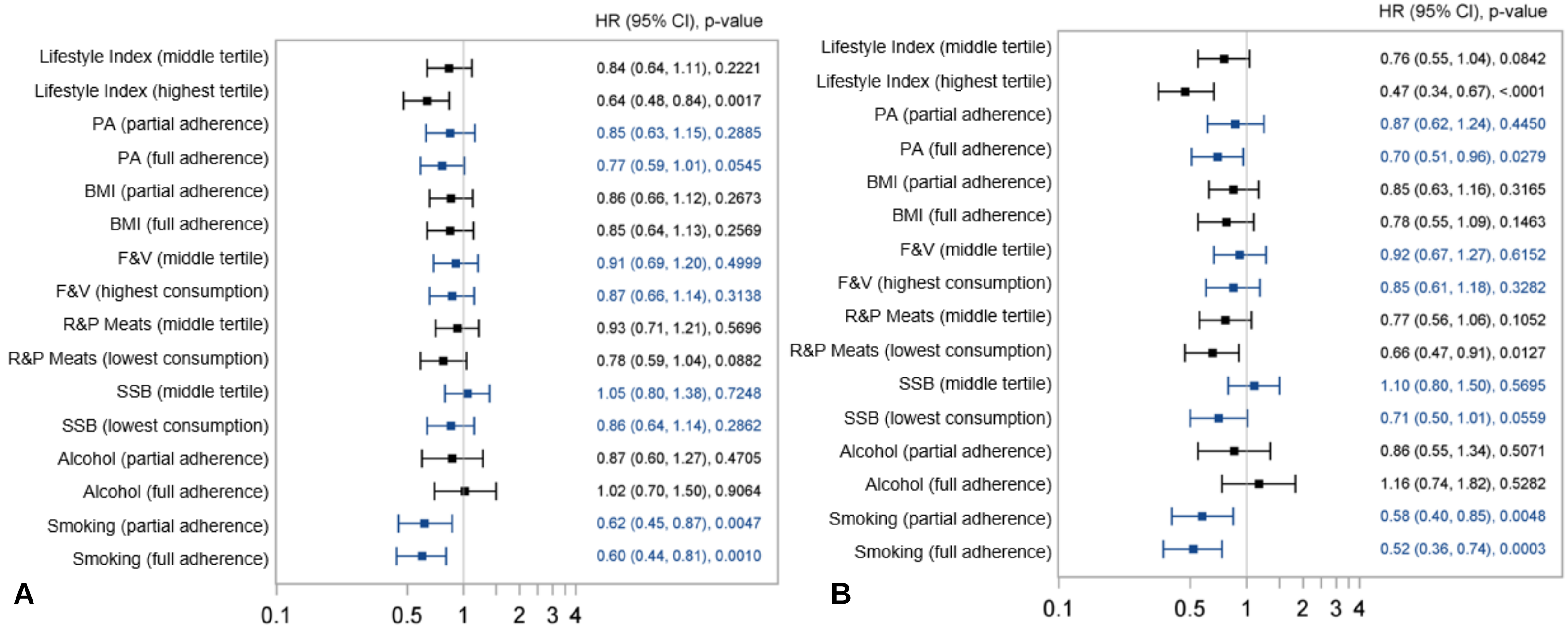

eFigure 2. Hazard Ratios (HR) and 95% Confidence Intervals (CI) Representing Associations of the Lifestyle Index Score (LIS) and Individual Lifestyles Before Diagnosis (Q1) With (A) Disease Recurrence and (B) Mortality in the DELCaP Study

For baseline (Q1) data, standard Cox models were used to estimate multivariable associations. The highest versus lowest LIS in the four weeks prior to study enrollment was associated with a 36% reduced hazard of recurrence and a 53% reduced hazard of mortality. Being a never or former smoker was associated with significant reductions in recurrence. Being a never- or former-smoker, meeting the physical activity Guidelines, and lowest consumption of red and processed meats were associated with significant reductions in mortality.

© 2023 Cannioto RA et al. *JAMA Network Open.*

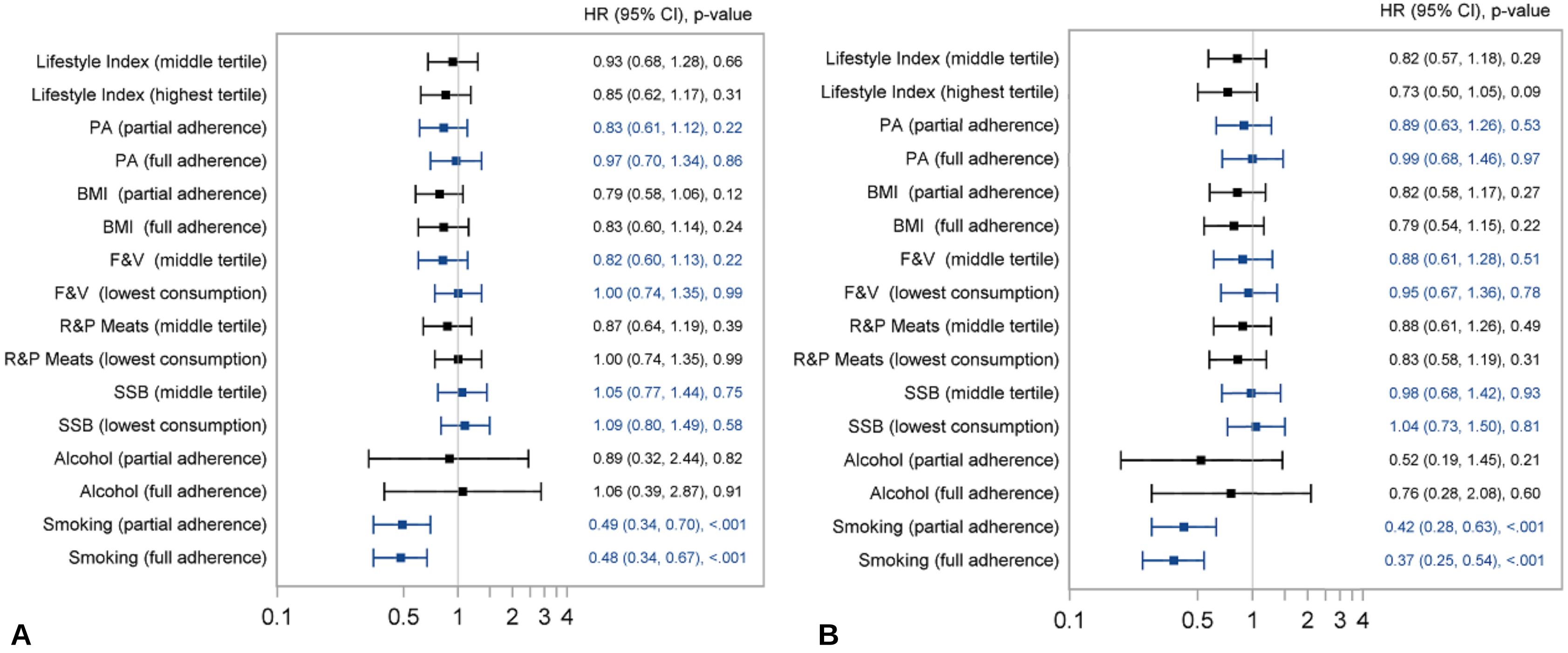

eFigure 3. Hazard Ratios (HR) and 95% Confidence Intervals (CI) Representing Associations of the Lifestyle Index Score (LIS) and Adherence to Individual Lifestyle Recommendations During Treatment (Q2) With (A) Disease Recurrence and (B) All-Cause Mortality in the DELCaP Study

For lifestyles at Q2, landmark analyses were incorporated to minimize the possibility of immortal time bias. Here, multivariable analyses show that adherence to the smoking recommendation was the only lifestyle factor associated with statistically significant improved outcomes.

© 2023 Cannioto RA et al. *JAMA Network Open.*

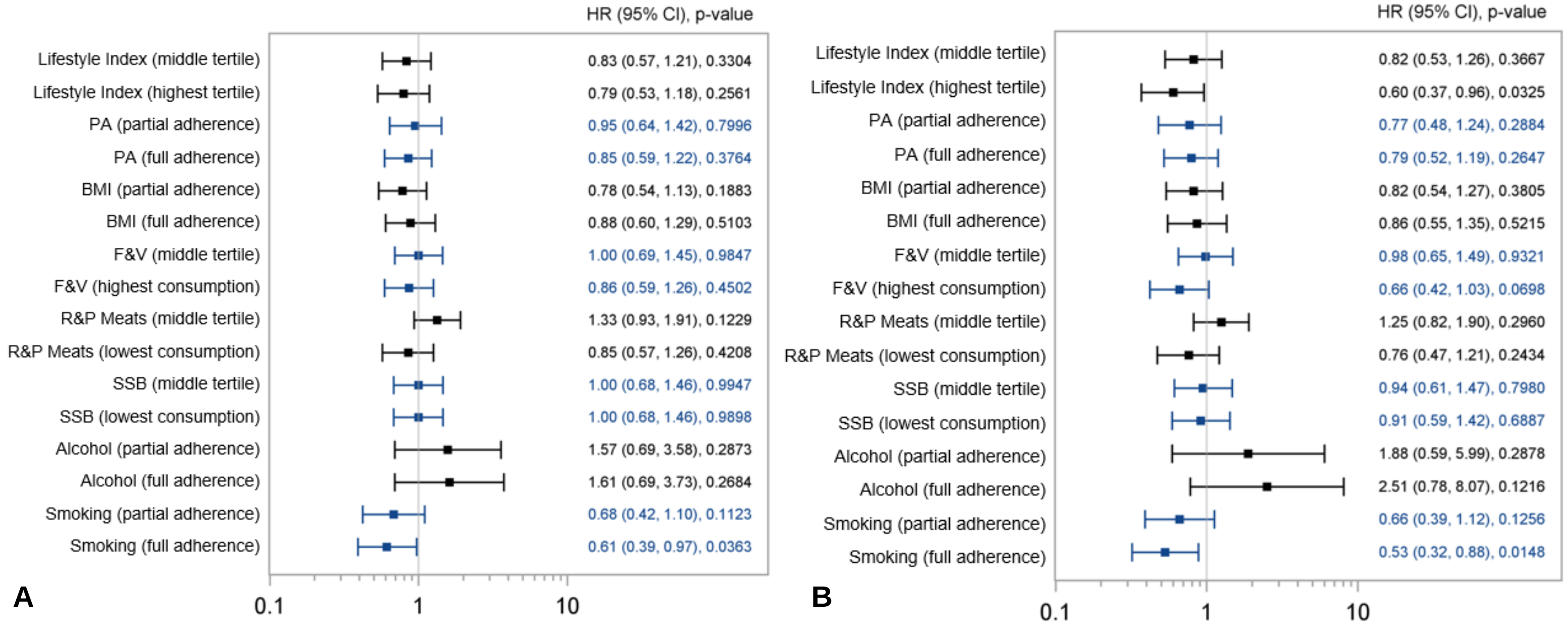

eFigure 4. Hazard Ratios (HR) and 95% Confidence Intervals (CI) Representing Associations of the Lifestyle Index Score and Adherence to Individual Lifestyle Recommendations at One-Year Follow-Up (Q3) With (A) Disease Recurrence and (B) All-Cause Mortality in the DELCaP Study

For lifestyles at one-year follow-up (Q3), landmark analyses were incorporated to minimize the possibility of immortal time bias. Here, multivariable analyses show that the highest versus lowest LIS at one year follow-up was associated with a 40% reduced hazard of mortality. Being a never-smoker was the only factor significantly associated with disease recurrence and mortality.

© 2023 Cannioto RA et al. *JAMA Network Open.*

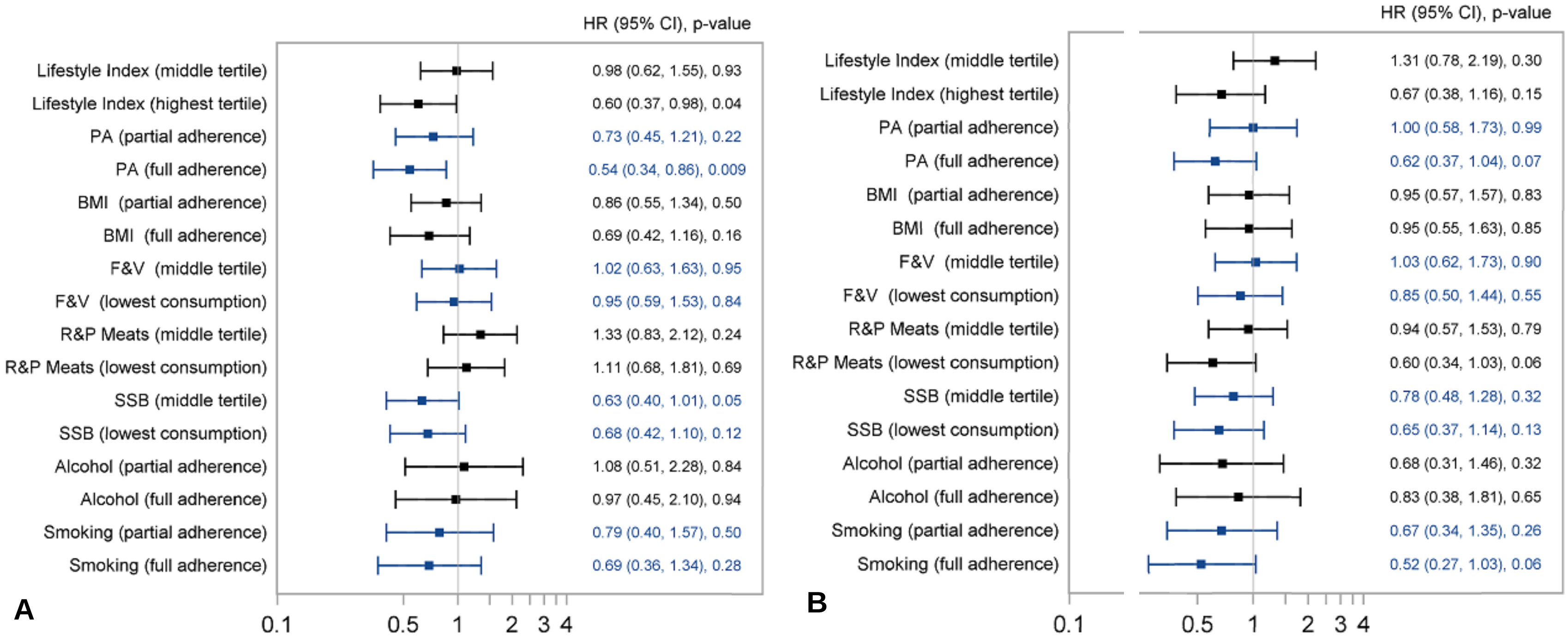

eFigure 5. Hazard Ratios (HR) and 95% Confidence Intervals (CI) Representing Associations of the Lifestyle Index Score and Adherence to Individual Lifestyle Recommendations at Two-Year Follow-up (Q4) With (A) Disease Recurrence and (B) All-Cause Mortality in the DELCaP Study

For lifestyles at two-year follow-up (Q4), landmark analyses were incorporated to minimize the possibility of immortal time bias. Here, multivariable analyses show that highest versus lowest LIS was associated with a 40% reduced hazard of disease recurrence. Physical activity was the only individual factor significantly associated with recurrence. Specifically, meeting the physical activity Guidelines was associated with a 46% reduced hazard of recurrence.

© 2023 Cannioto RA et al. *JAMA Network Open.*

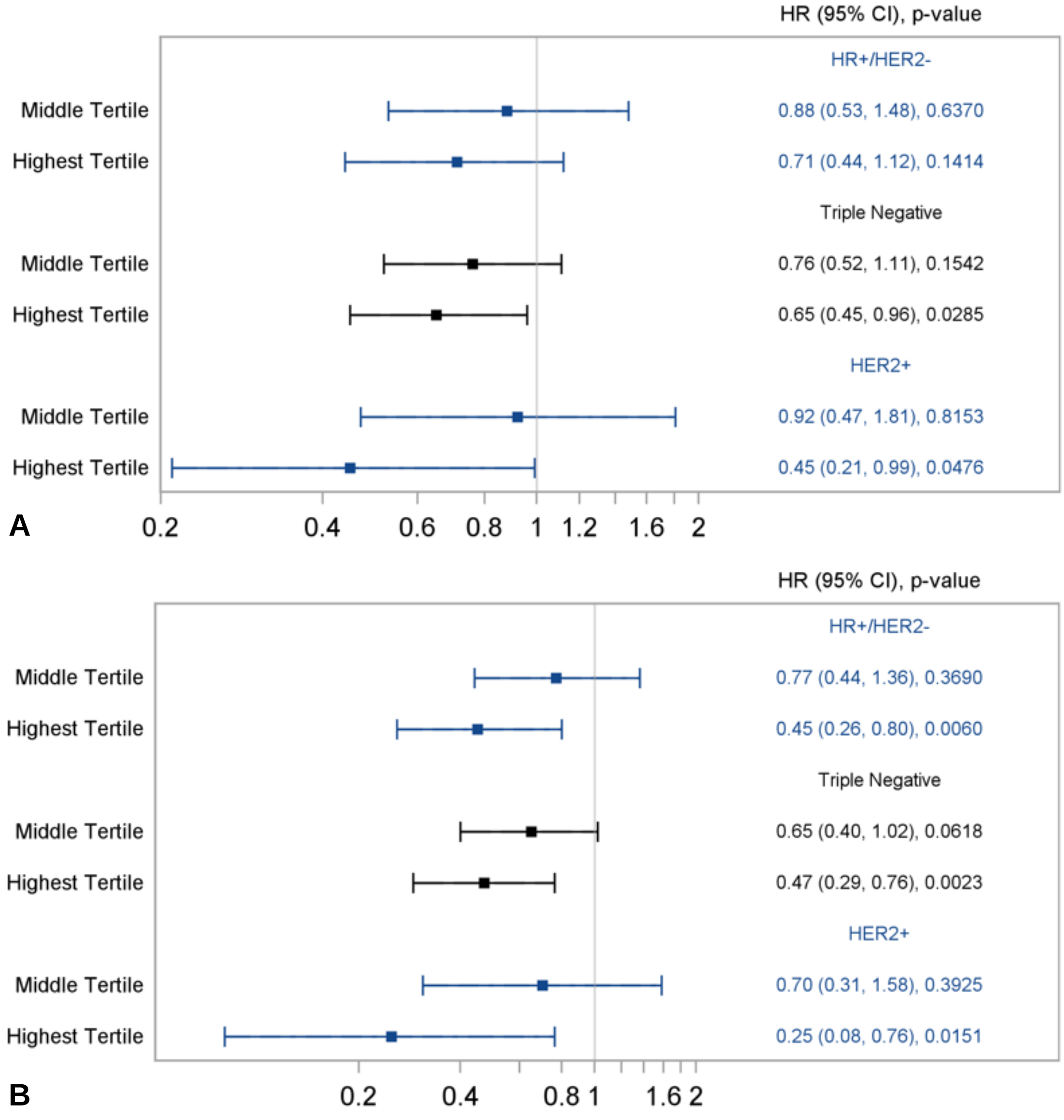

eFigure 6. Hazard Ratios (HR) and 95% Confidence Intervals (CI) Representing Associations of the Aggregate Lifestyle Index Score With (A) Disease Recurrence and (B) All-Cause Mortality According to Tumor Subtype in the DELCaP Study

Highest versus lowest Lifestyle Index Score was associated with significant reductions in disease recurrence for HER+ and Triple Negative BC (TNBC) and significantly reduced mortality for all subtypes.

© 2023 Cannioto RA et al. *JAMA Network Open.*

eReferences

1. Chasan-Taber L, Erickson JB, McBride JW, Nasca PC, Chasan-Taber S, Freedson PS. Reproducibility of a self-administered lifetime physical activity questionnaire among female college alumnae. *American journal of epidemiology.* 2002;155(3):282-289.
2. Ainsworth BE, Haskell WL, Herrmann SD, et al. 2011 Compendium of Physical Activities: a second update of codes and MET values. *Medicine and science in sports and exercise.* 2011;43(8):1575-1581.
3. Ainsworth BE, Haskell WL, Leon AS, et al. Compendium of physical activities: classification of energy costs of human physical activities. *Medicine and science in sports and exercise.* 1993;25(1):71-80.
4. Ainsworth BE, Haskell WL, Whitt MC, et al. Compendium of physical activities: an update of activity codes and MET intensities. *Medicine and science in sports and exercise.* 2000;32(9 Suppl):S498-504.
5. USDHHS. 2018 Physical Activity Guidelines for Americans. In. Washington, D.C.: Office of Disease Prevention and Health Promotion; 2018.
6. Cannioto RA, Dighe S, Mahoney MC, et al. Habitual recreational physical activity is associated with significantly improved survival in cancer patients: evidence from the Roswell Park Data Bank and BioRepository. *Cancer causes & control : CCC.* 2019;30(1):1-12.
7. O'Donovan G, Lee IM, Hamer M, Stamatakis E. Association of "Weekend Warrior" and Other Leisure Time Physical Activity Patterns With Risks for All-Cause, Cardiovascular Disease, and Cancer Mortality. *JAMA internal medicine.* 2017;177(3):335-342.
8. WHO. *Obesity: preventing and managing the global epidemic.* World Health Organization; 2000.
9. Satia-Abouta J, Patterson RE, King IB, et al. Reliability and validity of self-report of vitamin and mineral supplement use in the vitamins and lifestyle study. *American journal of epidemiology.* 2003;157(10):944-954.
10. Rothman KJ, Greenland S, Lash TL. Modern epidemiology. In: Vol 3. Philadelphia PA: Wolters Kluwer Health/Lippincott Williams & Wilkins Philadelphia; 2008:758.
11. Maldonado G, Greenland S. Simulation study of confounder-selection strategies. *American journal of epidemiology.* 1993;138(11):923-936.
12. Langston ME, Sfanos KS, Khan S, et al. Why Do Epidemiologic Studies Find an Inverse Association Between Intraprostatic Inflammation and Prostate Cancer: A Possible Role for Colliding Bias? *Cancer epidemiology, biomarkers & prevention : a publication of the American Association for Cancer Research, cosponsored by the American Society of Preventive Oncology.* 2021;30(2):255-259.
13. VanderWeele TJ, Ding P. Sensitivity Analysis in Observational Research: Introducing the E-Value. *Annals of internal medicine.* 2017;167(4):268-274.
14. Dekker FW, de Mutsert R, van Dijk PC, Zoccali C, Jager KJ. Survival analysis: time-dependent effects and time-varying risk factors. *Kidney international.* 2008;74(8):994-997.
15. Agarwal P, Moshier E, Ru M, et al. Immortal Time Bias in Observational Studies of Time-to-Event Outcomes: Assessing Effects of Postmastectomy Radiation Therapy Using the National Cancer Database. *Cancer control : journal of the Moffitt Cancer Center.* 2018;25(1):1073274818789355.

© 2023 Cannioto RA et al. *JAMA Network Open.*
