# Supplementary material for: Adherence to Cancer Prevention Lifestyle Recommendations Before, During, and 2 Years After Treatment for High-risk Breast Cancer
Source: JAMA Netw Open. 2023 May 4;6(5):e2311673. doi: 10.1001/jamanetworkopen.2023.11673 (PMC10160875; doi:10.1001/jamanetworkopen.2023.11673)
Supplement: Supplement 2. — Data Sharing Statement [file jamanetwopen-e2311673-s002.pdf]

Data Sharing Statement

Cannioto. Adherence to Cancer Prevention Lifestyle Recommendations Before, During, and 2 Years After Treatment for High-Risk Breast Cancer. *JAMA Netw Open*. Published May 04, 2023. doi:10.1001/jamanetworkopen.2023.11673

Data
Data available: Yes
Data types: Deidentified participant data, Data dictionary
How to access data: christine.ambrosone@roswellpark.org
When available: With publication

Supporting Documents
Document types: None

Additional Information
Who can access the data: Data will be made available to researchers whose proposed use of data has been approved
Types of analyses: Epidemiological investigations of the relationship between lifestyles and breast cancer survival
Mechanisms of data availability: Data will be made available upon approval of research proposal and with a completed data sharing agreement
